# Focal therapy of prostate cancer: Assessment with prostate-specific membrane antigen (PSMA) imaging

**DOI:** 10.1016/j.eucr.2023.102461

**Published:** 2023-06-09

**Authors:** Mark Topoozian, Jeremie Calais, Ely Felker, Anthony Sisk, Samantha Gonzalez, Sean J. Lee, Leonard S. Marks

**Affiliations:** a Department of Urology, David Geffen School of Medicine at UCLA, United States; b Department of Nuclear Medicine, David Geffen School of Medicine at UCLA, United States; c Department of Radiology, David Geffen School of Medicine at UCLA, United States; d Department of Pathology, David Geffen School of Medicine at UCLA, United States

**Keywords:** Prostate cancer, Focal therapy, PSMA, HIFU

## Abstract

Focal therapy of prostate cancer (PCa) is currently of great interest, but a metric of success. other than biopsy, is not yet available. In a patient with a repeatedly negative MRI and negative systematic biopsies, a scan employing the radioisotope ^68^Ga-PSMA-11 PET/CT identified a PSMA-avid hotspot in the prostate. PSMA-guided biopsy confirmed the diagnosis of a clinically-significant PCa. Following ablation of the lesion with high-intensity focused ultrasound (HIFU), the PSMA-avid lesion disappeared and targeted biopsy confirmed a fibrotic scar with no residual cancer. PSMA imaging may have a role in guiding diagnosis, focal ablation, and follow-up of men with PCa.

## 1 Introduction

Focal therapy of prostate cancer (PCa) (i.e., partial gland ablation, PGA) offers the potential for disease eradification without the after-effects of surgery or radiation. Candidates for focal therapy are selected on the basis of biopsy guided by magnetic resonance imaging (MRI). However, in some 15%of cases MRI may not reveal the PCa.^1^ In this report, a man with a falsely negative MRI underwent imaging with a different modality, radioisotope scanning with prostate-specific membrane antigen (PSMA, ^68^Ga-PSMA-11 PET/CT).^2^ PSMA-imaging was used to guide biopsy-detection of the PCa; to guide treatment of the cancer focally with high-intensity ultrasound (HIFU); and to show treatment success in follow-up, suggesting a possible new application for PSMA imaging.

## 2 Case Presentation

**Fig. 1:**
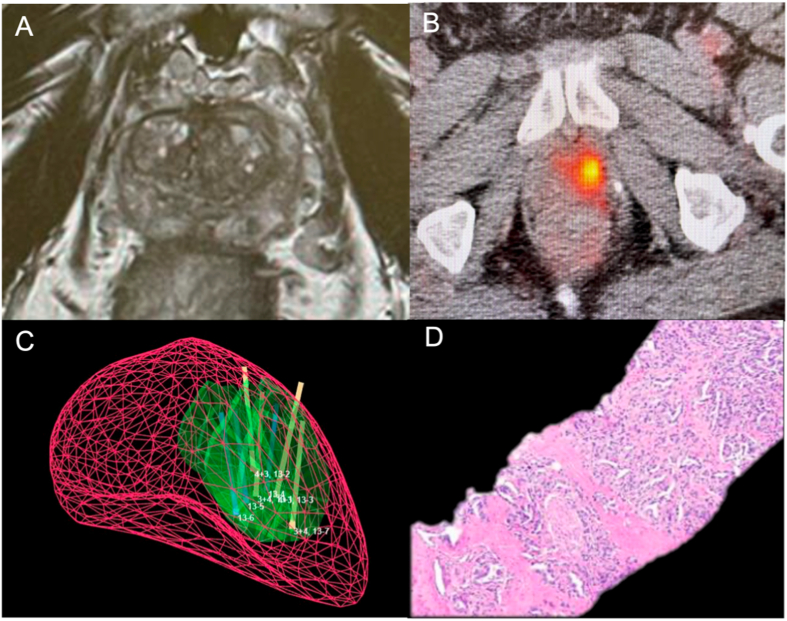
Pre-treatment images. (A) Prostate MRI (T2 weighted image) shows no lesions; three prior sets of systematic biopsies were all negative. (B) PSMA scan shows intense focal uptake in left anterior prostate (yellow spot), SUV max = 5, Primary Score = 4. (C) images from screen of fusion device (Artemis) during prostate biopsy (PSMA PET-CT/US) show contoured region of interest, ROI (green) and targeted biopsy cores (tan). (D) targeted biopsy core reveals Gleason Score 4 + 3 = 7 (GGG-3) (H&E, ×10). (For interpretation of the references to colour in this figure legend, the reader is referred to the Web version of this article.)

Because of a rising serum PSA level, a 75 y.o. Caucasian male underwent an ultrasound-guided prostate biopsy in February 2020. One of the 12 random samples contained a 1.5 mm focus of PCa (GG3). Subsequent to the biopsy a mpMRI of the prostate was performed, revealing no abnormalities (prostate volume = 31 cc). Patient deferred treatment, and PSA level continued to escalate. A second MRI was performed in June 2021; it was also negative. A second systematic biopsy, employing template guidance, was performed in July 2021 (18 cores); it was also negative. PSA continued to escalate, and in November 2021 a third MRI was performed; again, the MRI showed no abnormalities (Fig. 1A).

As part of an ongoing clinical trial evaluating PSMA for biopsy guidance (NCT05160597), patient underwent In December 2021 a PSMA PET/CT scan, revealing a region of PSMA avidity in the left mid-gland (SUV = 5) (Fig. 1B). The PSMA focus was contoured (J.C.) and the images were fused with real-time ultrasound using an Artemis device (Fig. 1C). Targeted biopsy, guided by PET-CT/US fusion, revealed four cores of PCa GG2 and GG3 in the PSMA ‘hotspot’ in the left mid-prostate (Fig. 1D). Serum PSA level at time of PSMA-guided biopsy was 6.9 ng/ml.

**Fig. 2:**
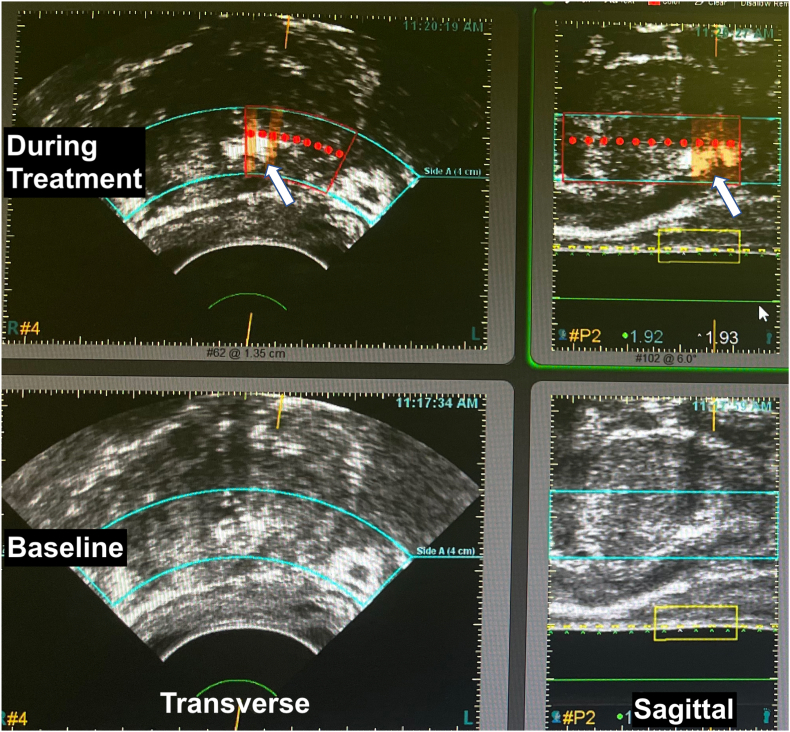
Images from screen of HIFU device (Sonablate 500) during focal therapy (partial gland ablation of left-sided tumor). Lower panel shows prostate before treatment started; upper panel shows treatment in progress; left side, transverse images; right side sagittal. PSMA hotspot (see Fig. 1) helped provide cognitive co-registration. Red dots indicate HIFU treatment foci at mid-zone of prostate. Note ‘popcorn’ effect (arrows), indicating cavitation created by treatment. (For interpretation of the references to colour in this figure legend, the reader is referred to the Web version of this article.)

A left PGA using HIFU (Sonablate 5000) was performed in March 2022 (Fig. 2). The procedure employed approximately 27k joules delivered in 3 planes, over a 2-h treatment time. Visual reference to PSMA lesion was used to help determine margins of treatment.

**Fig. 3:**
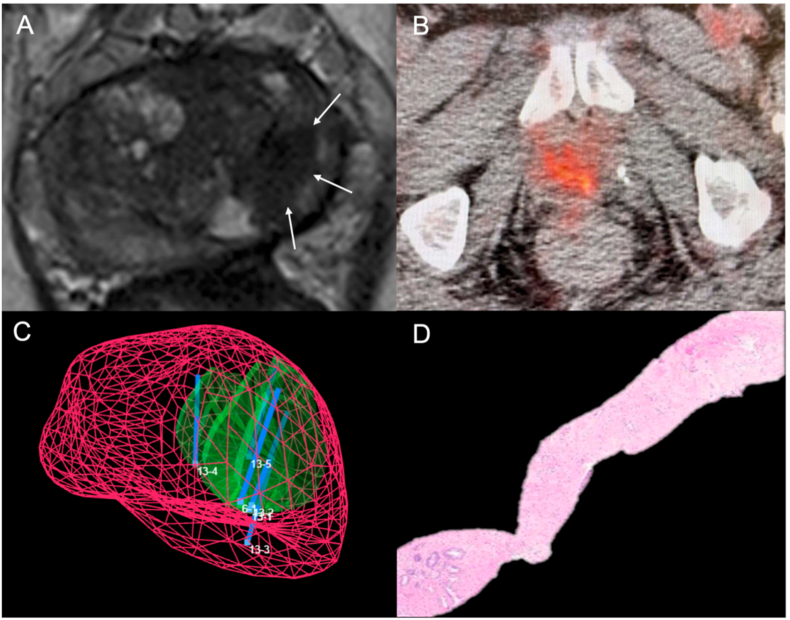
Follow-up MRI in October 2022 showed treatment effect and no suspicious regions (Fig. 3A). PSMA scan at that time showed disappearance of the PSMA hotspot (Fig. 3B). Biopsy targeting the site of the previous PSMA hotspot and adjacent left lobe (Fig. 3C) revealed only fibrosis upon pathologic analysis (Fig. 3D). One year post-treatment PSA is 0.7 ng/ml, and urinary and sexual function are unchanged from pre-treatment status (using alfuzosin and sildenafil as before).

Follow-up MRI in October 2022 showed treatment effect and no suspicious regions (Fig. 3A). PSMA scan at that time showed disappearance of the PSMA hotspot (Fig. 3B). Biopsy targeting the site of the previous PSMA hotspot and adjacent left lobe (Fig. 3C) revealed only fibrosis upon pathologic analysis (Fig. 3D). One year post-treatment PSA is 0.7 ng/ml, and urinary and sexual function are unchanged from pre-treatment status (using alfuzosin and sildenafil as before).

## 3 Discussion

A PSMA-based imaging agent (^68^Ga-PSMA-11) was first cleared by the U.S. FDA in December 2020. Six months later a commercially-available agent (^18^F-PSMA-1007) was also cleared.^2^ Approved indications for both PSMA forms are (1) staging of PCa and (2) detection of recurrent PCa after definitive primary treatment. The present report confirms the work of Emmett^3^ and others, showing that PSMA uptake can reveal localized cancer within the prostate and serve as a biopsy target when MRI fails to disclose the lesion. This report further shows that resolution of PSMA uptake after ablation may, at least in the near term, be an indicator of successful prostate focal therapy.

Interest in prostate focal therapy, which is an offshoot of targeted biopsy, is growing rapidly. Many newly-diagnosed cases of prostate cancer are potential candidates for focal therapy. Patient demand for focal therapy is increasing dramatically. However, a metric for treatment success---other than biopsy---has yet to emerge. We and others have shown that after focal therapy, serum PSA levels may decline and MRI-visible lesions may disappear, but within prostate tissue, i.e., upon biopsy, cancer can still be present.^4^

Burger and associates showed that after focal HIFU, MRI studies are often falsely negative, i.e., persistent cancer is not revealed by MRI.^5^ The Burger findings accord with a large study of focal cryotherapy in which MRI lesions disappeared after treatment in 74% (96/130) of patients, yet 23% of them (22/96) had persistent csPCa upon biopsy.^4^ The present case further shows how PSMA uptake reflects both presence (at diagnosis, Fig. 1B) and absence (after successful ablation, Fig. 2B) of clinically-significant PCa.

We and others have shown that biopsy is currently required to diagnose and confirm successful ablation. A non-invasive method to diagnose, evaluate and follow patients undergoing prostate focal therapy would be highly desirable. The cancer-specificity of PSMA uptake in the prostate---when SUV scores are high**---** lends promise to the new diagnostic. The clinical trial NCT05160597, when completed, may clarify a role for PSMA scanning in diagnosis and follow-up of men undergoing focal therapy of prostate cancer.

## Author contributions

Conceptualization (Marks, Calais); Data curation (Topoozian, Felker, Gonzalez, Sisk, Marks); Formal analysis (Gonzalez, Calais, Marks, Topoozian); Funding acquisition (Marks, Calais); Investigation (Marks, Topoozian); Methodology (Marks, Calais, Lee); Writing, editing (Marks, Calais, Topoozian). Corresponding author: Dr. Marks (lmarks@mednet.ucla.edu).

## Funding Acknowledgement

Dr. Marks’ work supported in part by 10.13039/100000054National Cancer Institute, (R01CA195505); and 10.13039/100016206UCLA CTSI, Grant/Award Number: UL1TR000124. PSMA scanning donated by Prof. Johannes Czernin, UCLA Nuclear Medicine Dept.
